# New York vs. Philadelphia

**Published:** 1859-11

**Authors:** 


### *New York* vs. *Philadelphia.*

The following from the Philadelphia Medical & Surgical Reporter, shows the rivalry existing between these two great cities. A good medical education can doubtless be obtained either in Philadelphia or New York, but these are not the only cities in which medicine is successfully taught, though our cotemporaries doubtless so think while they do not say so in so many words. But Western and Southern students have learned from experience, that they can be as well and as thoroughly instructed in their profession at home, as in either of these rival cities, and hence they naturally prefer to encourage their own institutions.

"The New York Medical Press publishes a long article copied from a Philadelphia newspaper, showing the acknowledged commercial superiority of New York. This is offered as an argument as to the attractiveness of that city to medical students. But, fortunately, students in seeking a medical education, desire neither to buy, sell, or *be sold.* New York is a great city of shop-keepers, with a dense population, packed into about one-third of the space of Philadelphia, and crowded into less than half the number of dwelling-houses. —*St. Louis Medical and Surgical Journal.*

